# New York Odontological Society

**Published:** 1886-04

**Authors:** 


### NEW YORK ODONTOLOGICAL SOCIETY.

REPORTED EXPRESSLY FOR THE INDEPENDENT PRACTITIONER.

The regular monthly meeting of this society was held in the parlors of the New York Academy of Medicine, on Tuesday evening, March 9th, the President, Dr. E. A. Bogue, in the chair.

Prof. R. Ogden Doremus addressed the society upon the subject of "Cocaine and its Effects." The professor considered cocaine a marvelous drug, and its effects on the animal economy of a peculiar and sometimes of a startling character He felt like saying a few words to "sound the alarm" concerning the indiscriminate employment of this agent, which he classed among poisons, and which he thought should be so labeled by whoever dispensed it. Like arsenic, the symptoms following its use were exceedingly varied and equally confusing. In overdoses it sometimes causes giddiness, vomiting, spasmodic muscular action, accelerated pulse, flushed face, dilated pupils, salivation, neuralgia, and paralysis. The unpleasant effects it produces sometimes last half an hour, and sometimes a very much longer period. It should be used with very great caution, and for hypodermic injections the quantity should never exceed five or six minims of a four per cent. solution. The doctor read several letters from physicians giving reports of cases where very unpleasant effects were attributed to what had been denominated "Cocaine poisoning."

Although the elements forming the alkaloid were simple, viz: carbon, oxygen, nitrogen, etc, they were, nevertheless, the same that formed the base of other powerful or poisonous alkaloids. It is the peculiar combination of these elements that give them their peculiar properties, but how or why is yet a mystery. The professor spoke of experiments on some of the lower order of animals, where cocaine had caused salivation or death.

Dr. Hoyt, a New York physician, to whom one of the letters read by Prof. Doremus referred "as a sufferer from cocaine poisoning," being present at the meeting, was invited by the president to state his experience as a patient under the influence of this drug. Doctor Hoyt stated that he called on a prominent dentist in the city some eighteen or more months ago, for the purpose of having a tooth filled. It was a right inferior incisor, with a cavity extending far down on the cervical border, and which proved exceedingly sensitive to the touch of an excavator. In order to lessen the pain, cocaine was applied to the cavity some half dozen times at intervals, and he imagined with a sl ght degree of benefit, yet the operation gave him intense pain, despite the applications. After completing the preparation of the cavity, so far as was possible under the circumstances, the dentist inserted a gold filling which, however, from insufficiency of anchorage, came out in process of finishing, and a filling of oxy-phosphate of zinc was substituted. On the following morning Dr. Hoyt experienced difficulty in efforts to swallow his food, having lost the power of deglutition, and the right side of his face had a strange or benumbed feeling, which continually became more marked for two days, when the muscles of the face on that side, and of the arm, became partly paralyzed. This condition lasted ten days, after which time signs of improvement were manifested. Electricity was employed, and other means resorted to to effect relief, but eight weeks elapsed before a cure was effected. Even for a year after, the tooth and face were sensitive to thermal influences.

The doctor is a hale and robust specimen of the *genus homo*, and seemed to take pride in saying that he had never before suffered from any physical ailment. He believes his trouble in this instance due in part to the excessive pain caused by the operation, giving such irritation to the tooth-pulp as to affect the nerves of the face, and in part to the application of the drug, which also had a specific action on these nerves.

In reply to inquiries concerning exposure of the pulp, Dr. Hoyt stated that he did not believe the pulp was fully exposed, but nearly so.

*Dr. C. A. Woodward*—Asked Prof Doremus if any antidote for cocaine poisoning had been discovered; to which query Prof. D. said he thought not.

Dr. Hoyt believed nux vomica an antidote. He used it in his own case.

*Dr. Bogue*—Remarked that from a hypodermical injection in his own mouth he experienced at first a degree of nausea, then a feeling of numbness of the face, which lasted twenty-four hours.

Dr. E. H. Raymond read a paper giving what he knew of the effects of cocaine from his personal observation. Although he had read of some cases of partial paralysis resulting from the use of this drug, he was, nevertheless, not afraid of it, yet admitted that it should be used intelligently and with due caution. Wishing to avoid giving pain in preparing cavities in sensitive teeth, he was glad to find some agent that would tend to render such operations painless. Various methods for producing local anæsthesia had been tested, but most of them were unsatisfactory, Dr. Raymond gave a somewhat lengthy recital of cases he had treated by hypodermic injections of cocaine, and of the results in each case. He also spoke of the various temperaments of patients thus treated, giving emphasis to the fact that temperament had much to do in giving direction to results, and it is a matter of great importance that the temperament of patients under any sort of treatment should be duly considered. As to whatever may be said concerning cocaine and its effects, he believed that "conservatism is better than enthusiasm." The doctor cited cases of mistaken diagnosis, and depicted the dangers liable to result from such mistakes ; also reminded his hearers that newspaper reports of surgical cases are not always accurate or reliable, and that due allowance should be made for exaggeration. He referred to the general and free use of cocaine among the natives of countries where it is gathered, and its harmless effects on those who partake of it. He thinks we should deal with facts and not be blinded by speculations, and so check the wheels of progress. If any remedial agent is suspected of producing injury, heaps of condemnation are cast upon it. If a cure happens to be effected by its use the remedy is supposed to be all right, but if death follows it is all wrong.

From the doctor's closing remarks one might infer that many individuals are much influenced by fancies and prejudices.

*Dr. J. W. Clowes*—Could see no necessity for using cocaine as an obtunder of sensitive dentine. He preferred chloroform, which he conveys to the cavity on a pellet of cotton, even allowing a little of it to be swallowed by the patient. With him it acts like a charm. He favors the practice of killing pulps with a paste of arsenic and creosote, but says that morphine should not be added, as it counteracts the action of the arsenic. Dr. Clowes intimated that suggestions from old heads were worthy of consideration.

*Dr. G. W. Weld*—Claimed that a spray of the solution of muriate of cocaine forced against the roof of the mouth was of great benefit to the dentist in enabling him to take impressions for artificial appliances. In five minutes after using it impressions could be taken of the most sensitive mouth without trouble, and with no harm whatever to the mucous membrane. Dr. Weld exhibited two styles of atomizer, either of which would answer the required purpose.

*Dr. N. W. Kingsley*—Knew of something far better than a spray of cocaine, claiming that skill was all that was needed.

*Dr. J. Morgan Howe*—Thinks cocaine may be of use for some purposes and useless for others. For sensitive dentine he thinks it not worth the while to employ it. He objects to Dr. Raymond's method of injecting it into the mental foramen, which he considers too much risk for so little gain. It may, however, be used advantageously as an application to the gums when removing calculus, opening abscesses, probing sinuses, etc. At the risk of being considered by Dr. Kingsley "unskilled," he had been much aided by the use of cocaine in taking impressions of the mouth. Dr. Howe cited one case in particular, which was quite interesting. In endeavoring to get an impression of a gentleman's mouth for an artificial denture, he found the mucous membrane of the roof so exceedingly sensitive that it would not bear the touch of the impression cup, and even a touch with the finger caused severe gagging. The gentleman said that repeated efforts had previously been made by other dentists to get impressions of his mouth, but without success. He had submitted to trials nine different times, and each time he nearly gagged himself to death. Remembering that Dr. Bogue once suggested the use of cocaine in such cases, Dr Howe applied a little of the solution to the roof of the mouth, and in six minutes after took the impression without trouble.

*Prof. Doremus*—Did not wish it understood that he discountenanced the use of cocaine, but he thought it should always be employed with caution, and he objected to its being sold and used indiscriminately. He would have druggists who sell it label it "poison."

The President announced that on this occasion the order of business had been reversed in order to allow guests who had other engagements to depart early. He then called for "Incidents of office practice."

*Dr. C. D. Cook*—Presented a plaster model of a peculiar case, representing an upper set of natural teeth in which were six well developed bicuspids.

*Dr. J. M. Brigiotti*—(formerly of Paris,) sent to the society casts representing the teeth of a lady thirty-two years of age, where an unsightly and complicated articulation coupled with "jimber-jaw" was corrected, and this accomplished by extraction and a regulating appliance. In this case very good results were secured. Accompanying the casts was a short paper descriptive of the case and its treatment, which was read by the secretary.

